# Analysis of Residual Compressive Strength in 3D Four-Directional Braided Composites After Hygrothermal Aging

**DOI:** 10.3390/ma18061368

**Published:** 2025-03-20

**Authors:** Yongxin Niu, Lingze Bu, Shi Yan, Songming Cai, Zixiang Meng

**Affiliations:** Department of Engineering Mechanics, Harbin University of Science and Technology, Harbin 150080, China; niuyongxin2024@163.com (Y.N.); 18641440516@163.com (S.C.); meng_zixiang2022@163.com (Z.M.)

**Keywords:** three-dimensional four-directional braided composite materials, hygrothermal aging, compression after wet heat aging, failure mode, finite element simulation

## Abstract

This study investigates the effect of hygrothermal environments on the compressive properties of three-dimensional four-directional braided composites through experiments and finite element simulations, revealing the degradation behavior under various hygrothermal conditions. The results indicate that the moisture absorption behavior of the material conforms to Fick’s law. The longer the hygrothermal aging duration and the higher the temperature, the more significant the reduction in compressive performance, as evidenced by the continuous decline in ultimate stress. The hygrothermal environment primarily affects material performance through moisture absorption and thermal expansion characteristics of the epoxy resin, while the carbon fibers exhibit high stability in such conditions, maintaining the integrity of the three-dimensional four-directional structure. Microscopic observations reveal that hygrothermal aging exacerbates damage at the resin–fiber interface, leading to more pronounced stress concentration. Finite element simulations further quantify the internal stress distribution under hygrothermal conditions, demonstrating that moisture-induced expansion stress is more significant than thermal expansion stress, providing theoretical support and design guidance for improving the performance of composites in extreme environments.

## 1. Introduction

As an advanced material, three-dimensional woven composites have found significant applications in various industrial fields [1,2,3,4,5,6]. According to market research reports, due to their excellent mechanical properties, the market share of three-dimensional woven composites in aerospace, automotive, and shipbuilding industries has been growing annually. Examples include the wing beams of the Boeing 787 and thermal protection materials for spacecraft. It is expected that by 2025, the global aerospace composites market will reach $6 billion, with three-dimensional woven composites holding a significant position. They have now become core materials in high-tech industries [7,8,9]. Three-dimensional woven composites, as core materials in high-tech fields, often face extreme temperature and humidity conditions [10,11]. The synergistic effect of environmental temperature and humidity significantly affects the mechanical properties of the material and may lead to its degradation over time. Therefore, hygrothermal aging has become a key factor in assessing the long-term performance and durability of composites [12,13,14]. By exposing the material to controlled hygrothermal conditions in experiments, a deeper understanding of its performance in practical applications can be obtained, ensuring its reliability and extending its service life. Three-dimensional four-directional braided composites are composed of a resin matrix and fibers, which are tightly bonded through complex physical and chemical interactions. When composite materials are exposed to hygrothermal environments for extended periods, moisture absorption causes the resin matrix to expand, generating interfacial stresses that weaken the bond between the fibers and resin, thereby reducing the material’s strength. Furthermore, the combined effects of increased temperature and humidity induce inconsistent expansion between the resin matrix and fibers, increasing internal stress and further degrading the material’s performance. The hygrothermal environment also exacerbates the degradation of the fiber–resin interface, reducing the interfacial bond strength, which significantly lowers the compressive, tensile, and shear strengths of the composite material. Monitoring these changes is crucial for evaluating the long-term reliability of the material in extreme environments. Yu Kui et al. [15] conducted experimental studies on the performance of epoxy resin-based materials for repairing and reinforcing faulted rock formations and evaluated their penetration capabilities and strength-enhancing effects under different temperature conditions (0 °C, 15 °C, and 20 °C). Amoushahi et al. [16] studied the effects of temperature and humidity on the free vibration frequency and buckling load of composite laminates. Kumar et al. [17] analyzed the mechanical performance changes of carbon fiber-reinforced epoxy laminates in humid environments by controlling hygrothermal aging time, revealing that humid conditions cause varying degrees of reduction in longitudinal tensile strength, transverse tensile strength, and in-plane shear modulus. Experiments by Kawai et al. [18] demonstrated that, under normal temperature conditions, the fatigue strength of wet-woven carbon/epoxy laminates is approximately 11% lower than that of dry specimens. A.P. Chakraverty et al. [19] found that, under both hygrothermal and hydrothermal conditions, exposure time plays a critical role in the mechanical stability of glass fiber-reinforced epoxy composites (GRE). Benkhedda et al. [20] proposed a multi-scale transient hygrothermal analysis method that integrates micro- and macro-level analyses, enabling rapid calculation of the degradation in strength and stiffness of composites under hygrothermal environments. Alessandro Magazzù et al. [21] explored how nanotechnology tools such as Atomic Force Microscopy (AFM) and Optical Tweezers (OT) can be used to study the mechanical properties of soft matter. Nanotechnology is similarly applicable in the study of composite materials. In this paper, Scanning Electron Microscopy (SEM) was used to observe the damage and fiber changes that occurred after compression, particularly focusing on the interface damage between the resin and fibers. The analysis of these microstructures provides a foundation for further nanomechanical research, particularly when evaluating the internal stress distribution and interface strength of the material. Furthermore, finite element analysis helps to understand the mechanical behavior of materials at the microscopic level, thereby providing theoretical support for nanomechanics. On the other hand, the unique structural characteristics of three-dimensional four-directional braided composites result in excellent compressive performance. Fang et al. [22] investigated the uniaxial compressive mechanical properties of the material through finite element analysis and found that different braiding angles have a significant impact on compressive failure. Zhu et al. [23] found through a parametric model that the compressive performance of three-dimensional four-directional braided composites is inversely proportional to the braiding angle and directly proportional to the fiber volume fraction. Most studies on the effects of hygrothermal environments on composites have focused on laminated materials, while research on the widely used three-dimensional four-directional braided composites is relatively limited. Existing studies on the compressive performance of three-dimensional four-directional braided composites mainly focus on the influence of braiding angles, with limited research addressing compressive performance after hygrothermal aging. This study investigates the compressive performance of three-dimensional four-directional braided composites after hygrothermal aging. Two hygrothermal conditions (water baths at 40 °C and 70 °C) and three aging durations (500 h, 1000 h, and 2000 h) were considered, these temperatures were selected based on typical environmental conditions that composite materials may encounter in real-world applications. In this case, 40 °C represents a moderate thermal environment, similar to those found in automotive engine compartments or outdoor working conditions. In contrast, 70 °C simulates the extreme environments encountered in high-performance aerospace applications or high-temperature conditions that may arise during severe thermal cycling. The aging durations of 500 h, 1000 h, and 2000 h were determined based on previous experimental results regarding the effects of hygrothermal aging on composite materials [24,25,26,27]. These conditions are widely used in the literature to simulate the typical service environments of such materials. Additionally, the water bath environment was set up to accelerate the experimental process, allowing for the testing of the material’s mechanical properties under extreme conditions to ensure its reliability and stability in such environments. The effects of different water bath conditions and aging durations on the compressive performance of the materials under quasi-static loading were explored, with an emphasis on analyzing the strength degradation after hygrothermal aging. A scanning electron microscope was used to observe changes in fibers after material failure. Based on the four-step braiding method and the model proposed by Zhu [23], an appropriate finite element analysis model was developed to simulate the diffusion of water molecules within the three-dimensional four-directional braided composites, predict the internal stresses induced by hygrothermal environments, and evaluate their impact on the compressive performance of the specimens.

## 2. Experimental Materials and Process

### 2.1. Experimental Materials

This experiment utilized three-dimensional four-directional braided composite plates manufactured by Hubei Feilihua Quartz Glass Co., Ltd. in Jingzhou, China. The yarn used was Toray T700-SC-12000-50B carbon fiber, and the matrix was TED-86 epoxy resin. Specific material parameters are shown in Table 1.

### 2.2. Damp Heat Aging Test

Before the hygrothermal experiment, the specimens were cleaned with deionized water and dried in a vacuum environment until reaching an engineering dry state. Subsequently, the hygrothermal aging experiment was conducted using a constant temperature and humidity water bath. To investigate the effects of temperature and aging duration in a hygrothermal environment on material properties, the experiment was conducted under two temperature conditions (40 °C and 70 °C) and three aging durations (500 h, 1000 h, and 2000 h). Additionally, one group of specimens was prepared under normal temperature and humidity conditions as a control group. To ensure the reliability of the data, three samples were prepared for each group, and the average value was used as the experimental data. The specimens after drying treatment were measured for their initial weight, denoted as $W_{0}$. Every 24 h, the specimens were removed, dried, and weighed, and the average weight of each group was recorded as $W_{t}$. The procedure was repeated until the experiment reached the predetermined duration. After the experiment was completed, the moisture absorption rate of the specimens was calculated according to Equation (1).

(1) $$M_{t}=\frac{W_{t}-W_{0}}{W_{0}}\times 100\%$$

In the formula, $M_{t}$ represents the weight gain rate (%) of the specimen at time *t*; $W_{t}$ is the mass of the specimen (g) at time *t* during hygrothermal aging;$W_{0}$ is the initial weight (g) of the specimen before hygrothermal aging.

### 2.3. Compression Experiment

**Figure 1:**
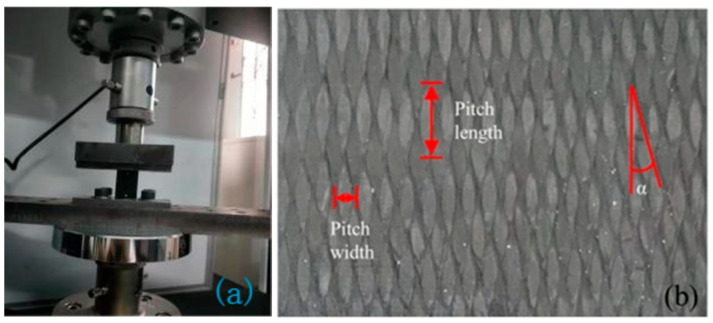
(**a**) Compression process of the specimen; (**b**) 3D braiding surface.

The compression test was conducted using a universal testing machine, model MTS2500, produced by MTS Systems Corporation, Eden Prairie, MN, USA, with a maximum load capacity of 1000 kN. This model can automatically generate a load–displacement curve, from which the stress–strain curve can be derived. The ultimate stress and Young’s modulus can be calculated from the curve, with the ultimate stress determined using Equation (2) and Young’s modulus calculated from the slope of the stress–strain curve. After the specimens were exposed to the specified hygrothermal environment and duration, a quasi-static compression test was performed. The material was placed at the center of the compression test platform to ensure it was subjected only to axial pressure. The loading speed was set to 0.5 mm/min, according to the standard. The compression process of the specimen is shown in Figure 1a:

(2) $$\sigma_{ultimate}=\frac{F_{\max}}{A}$$

where $\sigma_{ultimate}$ represents the ultimate failure stress, $F_{\max}$ is the maximum load that the specimen can withstand, and A denotes the contact area of the specimen.

## 3. Finite Element Analysis

### 3.1. Geometric Modeling

Three-dimensional four-directional braided composites are composed of tightly bound fiber bundles and matrix material. For finite element analysis of the fiber bundles, a four-step braiding method was employed. The yarns were arranged by carriers according to the cross-sectional shape of the preform and interlaced based on specific rules to complete the braiding process. The braided structure of the yarns is determined by the motion trajectory of the carriers. The carriers are distributed and moved on to the braiding machine according to specific rules, forming different arrangements and motion patterns. Here, α represents the angle between the yarn and the longitudinal axis. To more accurately simulate the diffusion of water molecules in three-dimensional four-directional braided composites under hygrothermal conditions and the resulting internal stress, a high fiber volume fraction model was established based on the parametric model proposed by Zhu [23].

(3) $$D_{y}=\sqrt{\frac{4\lambda }{\pi \rho }}$$

(4) $$h=\sqrt{\frac{\pi \sqrt{1+2\tan^{2}\alpha }}{V_{f}\cdot \tan^{2}\alpha }}D_{y}=\frac{W_{i}}{\tan\alpha }$$

(5) $$W_{i}=\sqrt{\frac{\pi \sqrt{1+2\tan^{2}\alpha }}{V_{f}}D_{y}}$$

where $D_{y}$ represents the equivalent diameter of the yarn, λ represents the yarn density, ρ denotes the fiber density, $W_{i}$ is the step distance of the carrier in the row or column direction, and h is the height increment of the braided structure after one step of row or column movement by the carrier. Table 2 shows the specific parameters calculated by Equations (3)–(5).

**Figure 2:**
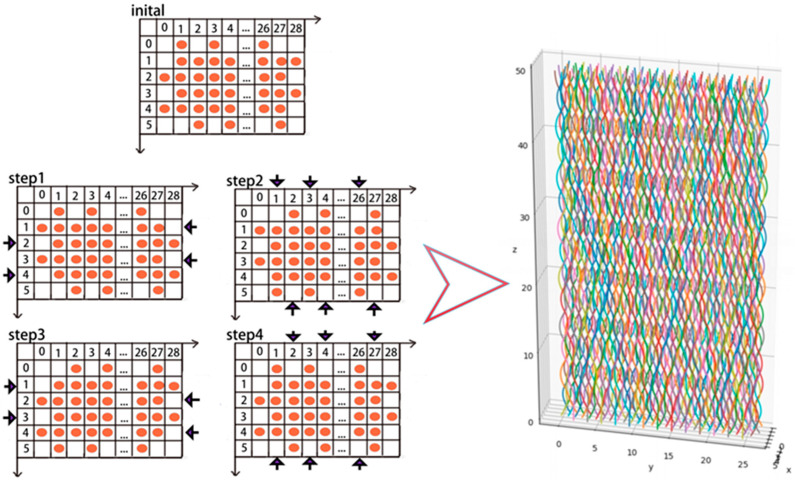
Simulated Yarn Paths Based on the “Four-Step Method”.

Based on the theoretical formula above, the projection of the yarns on the x–y plane can be represented in a 6 × 29 matrix. The “orange” circles indicate the projection of the braided yarns on the x-y plane, and the “arrows” represent the movement direction of the yarns in each step of the “four-step method”. By using Python software for calculation, the path of each yarn can be obtained, as shown in Figure 2.

This method of establishing fiber bundles treats the fabrication process of fabric composites as a nonlinear solid mechanics problem, it ensures the uniform distribution of carbon fibers throughout the model and allowing the shape of the yarn and the “tightening” issue to be ignored. However, in this approach, the trajectory of the yarns along the z-axis forms a polyline. It is critically important to transform these into smooth curves without altering the fiber trajectory [29]. For this aspect of the study, this paper employs B-spline curves for interpolation, generating smooth B-spline curves. This interpolation method demonstrates good convergence and relatively accurate calculations, with cubic spline interpolation being sufficient to meet the requirements. The specific mathematical computation logic is as follows:

When *k* = 1(a zeroth-order B-spline), the basis function is a piecewise constant function:

(6) $$B_{i,0}(u)=\left\{\begin{array}{c} 1,u⋲[u_{i},u_{i+1}]\\ 0,else \end{array}\right.$$

When *k* > 1, the value of the basis function $B_{i,k}(u)$ is calculated recursively.

(7) $$B_{i,k}(u)=\frac{u-u_{i}}{u_{i+k-1}-u_{i}}B_{i,k-1}(u)+\frac{u_{i+k}-u}{u_{i+k}-u_{i+1}}B_{i+1,k-1}(u)$$

After smoothing the yarns, a solid model was generated by sweeping and imported into the commercial software Abaqus. The “embedded constraint” method directly associates the motion of the dependent element nodes with the master elements without explicitly defining constraint relationships, avoiding contact nonlinearity issues and improving computational efficiency. Therefore, the simulation of fiber bundles and matrix material can be combined using the “embedded constraint” method.

### 3.2. Analysis of Internal Stress Induced by Hygrothermal Environment

The internal stress induced by the hygrothermal environment can be divided into two parts [30]: thermal stress caused by high temperatures and moisture expansion stress resulting from material hygroscopicity. This paper calculates these two stress components separately and combines them to simulate the hygrothermal coupling effect. Firstly, in the analysis of thermal stress, the primary cause is the mismatch in thermal expansion coefficients between the fiber bundles and the resin matrix. Carbon fibers exhibit high thermal stability. Geng’s research [31] indicates that the T700 carbon fiber bundles aligned parallel to the axial direction display negative thermal expansion properties within the temperature range of [−150 °C, +150 °C], while epoxy resin is more sensitive to temperature changes. This disparity in thermal expansion induces stress concentration at the material interface, causing debonding between the matrix and the fibers, which leads to reductions in strength and stiffness. For finite element analysis of thermal stress, the “thermal conduction” analysis step in Abaqus is used. Boundary conditions are set based on experimental conditions: the bottom is fixed, while the other five faces allow heat flow to evenly diffuse into the material, The mesh attributes are set to “temperature-displacement coupling”, as mentioned earlier, carbon fibers exhibit excellent thermal stability, thus their thermophysical properties remain stable in finite element simulations. In contrast, epoxy resin is more sensitive to temperature variations, and therefore its thermophysical parameters differ between the 40 °C and 70 °C environments, and the thermal parameters of the materials are listed in Table 3.

For stress induced by a moist environment, the moisture absorption process of fabric-reinforced composites can be accurately simulated using finite element methods, and the internal stress after moisture absorption can be calculated. It is assumed that the absorption process of water is carried out by mass diffusion, regardless of surface evaporation and convection phenomena. In the Abaqus 2022 software, the “mass diffusion” module is used to simulate the diffusion of water molecules under hygrothermal conditions. However, this module cannot directly extract residual stress caused by the moist environment. Therefore, for stress due to moisture, the “thermal diffusion” module is employed to simulate the diffusion process of water molecules. By comparing the equilibrium of the hygrothermal balance equation, thermodynamic parameters are substituted for humidity parameters to compute the corresponding stress field.

For this hygrothermal equivalence principle, it is first necessary to analyze and compare the “thermal conduction” equation with the “mass diffusion” equation. According to Fourier’s law of heat conduction, the heat dQ passing through the area dS per unit time can be expressed as:

(8) $$dQ=-\kappa \nabla T\cdot \vec{n}\cdot dS$$

where κ represents the Thermal conductivity, ∇T represents the Temperature gradient, $\vec{n}$ represents the unit normal vector.

Considering the total heat passing through the closed surface *Φ* that encloses the region *Ω* during the time interval (*t*_1_, *t*_2_) is:

(9) $$Q_{out}=\int_{t_{1}}^{t_{2}}\int_{\Phi }\kappa \cdot \nabla T\cdot \vec{n}\cdot dS\cdot dt$$

The heat absorbed by the medium is equal to the change in heat within the volume:

(10) $$Q_{in}=\int_{\Omega }\rho c\frac{\partial T}{\partial t}dV$$

where *c* represents the specific heat capacity of the medium, ρ represents the density of the medium.

According to the principle of energy conservation, the absorbed heat should equal the heat that flows out:

(11) $$\int_{\Omega }\rho c\frac{\partial T}{\partial t}dV=-\int_{\Omega }\nabla \cdot (\kappa \cdot \nabla T)\cdot dV$$

According to Gauss’s divergence theorem, the above equation is transformed into its differential form as:

(12) $$\rho c\frac{\partial T}{\partial t}=\nabla \cdot (\kappa \cdot \nabla T)$$

Similarly, based on Fick First Law, the diffused mass *dM* passing through the area *ds* per unit time is expressed as:

(13) $$dM=-D\cdot \nabla U\cdot \vec{n}\cdot dS$$

where *D* represents the Diffusion coefficient, ∇U represents the Concentration gradient.

For a region *Ω* enclosed by a closed surface *Φ*, the diffusion mass through the surface in time (*t*_1_, *t*_2_) is:

(14) $$M_{out}=\int_{t_{1}}^{t_{2}}\int_{\Phi }D\cdot \nabla T\cdot \vec{n}\cdot dS\cdot dt$$

The mass change of the diffusion into the region is expressed as:

(15) $$M_{in}=\int_{\Omega }\frac{\partial U}{\partial t}dV$$

According to the law of mass conservation, the mass flowing in is equal to the mass increase caused by the change in concentration:

(16) $$\int_{\Omega }\frac{\partial U}{\partial t}dV=-\int_{\Omega }\nabla \cdot (D\cdot \nabla T)\cdot dv$$

The differential form is:

(17) $$\frac{\partial U}{\partial t}=\nabla \cdot (D\cdot \nabla T)$$

**Figure 3:**
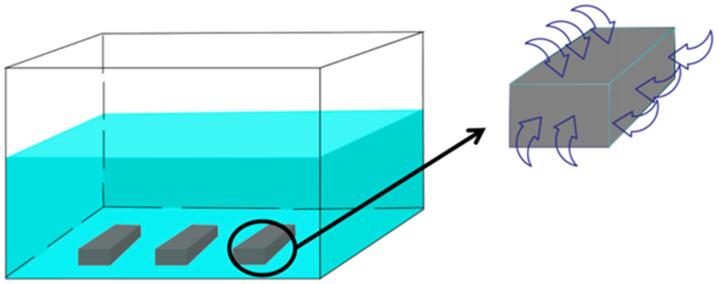
Humidity and heat experiment and boundary condition settings.

Through rigorous formula derivation, it is concluded that the two equation forms are similar, and the thermal conductivity and diffusion coefficient are equivalent parameters in a physical sense. Therefore, thermal diffusion and mass diffusion can be considered equivalent when calculating hygroscopic stress. In finite element analysis, the boundary condition is set such that the bottom of the material is fixed, and water molecules diffuse inward from the remaining five surfaces, according to this setup, the initial humidity field is considered to be uniformly distributed, the boundary conditions are shown in Figure 3. At this stage, the mesh attribute is set to “thermal diffusion” to simulate the humidity field of the material. Subsequently, the “humidity field” is used as a predefined field, and combined with the moisture expansion coefficient, the mesh attribute is adjusted to “three-dimensional stress” to extract the hygroscopic stress. The physical property parameters of carbon fiber and epoxy resin remain constant at the same water bath temperature. The specific equivalent relationship between “thermal diffusion” and “mass diffusion” is shown in Table 4. By coupling the “thermal stress” and “hygroscopic stress” caused by the hygrothermal environment, the hygrothermal stress of the material can be obtained through finite element simulation.

### 3.3. Compression Experiment and Failure Damage Criteria After Hygrothermal Aging

In the finite element simulation of the compression process after wet heat aging, the wet heat stress is taken as the initial stress field, and then the specimen compression simulation is carried out; in the simulation of compression experiments, the “Amplitude” option is set to allow the magnitude of the applied load to vary over time. The load is gradually increased to its maximum value rather than being applied instantaneously, thus achieving the effect of simulating quasi-static compression. To comprehensively and accurately study the damage and failure behavior of three-dimensional four-directional braided materials, the User-defined Material Subroutine is introduced. The matrix and fiber bundles are analyzed separately using the three-dimensional Hashin damage criteria [32]. For the failure modes of fiber bundles, axial tension, axial compression, transverse tension, and transverse compression are considered and evaluated based on the Hashin initial damage criteria. For the failure behavior of the resin matrix, the first strength theory is applied under both tensile and compressive conditions. The criteria formulas are as follows:

Fiber Bundle Axial Tension:

(18) $$d_{f1}^{T}={\left(\frac{\sigma_{11}}{F_{1}^{T}}\right)}^{2}+{\left(\frac{\sigma_{12}}{S_{12}}\right)}^{2}+{\left(\frac{\sigma_{13}}{S_{13}}\right)}^{2}\geq 1,\sigma_{11}>0$$

Fiber Bundle Axial Compression:

(19) $$d_{f1}^{c}={\left(\frac{\sigma_{11}}{F_{1}^{c}}\right)}^{2}\geq 1,\sigma_{11}<0$$

Fiber Bundle Transverse Tension:

(20) $$d_{f2}^{T}={\left(\frac{\sigma_{2}+\sigma_{3}}{F_{2}^{T}}\right)}^{2}+(\frac{\sigma_{23}^{2}-\sigma_{2}\sigma_{3}}{S_{23}^{2}})+{\left(\frac{\sigma_{12}}{S_{12}}\right)}^{2}+{\left(\frac{\sigma_{13}}{\sigma_{13}}\right)}^{2}\geq 1$$

Fiber Bundle Transverse Compression:

(21) $$\begin{array}{c} d_{f2}^{c}={\left(\frac{\sigma_{2}+\sigma_{3}}{{2S}_{12}}\right)}^{2}+\frac{\sigma_{2}+\sigma_{3}}{F_{2}^{c}}[{\left(\frac{F_{2}^{c}}{{2S}_{12}}\right)}^{2}-1]+(\frac{\sigma_{23}^{2}-\sigma_{2}\sigma_{3}}{S_{23}^{2}})\\ +{\left(\frac{\sigma_{12}}{S_{12}}\right)}^{2}+{\left(\frac{\sigma_{13}}{\sigma_{13}}\right)}^{2}\geq 1 \end{array}$$

Matrix Tensile Failure:

(22) $$d_{m}^{T}={\left(\frac{\sigma_{1}}{F_{m}^{T}}\right)}^{2}={\left(\frac{\sigma_{1}}{F_{1}^{T}}\right)}^{2}\geq 1,\sigma_{1}\geq 0$$

Matrix Compressive Failure:

(23) $$d_{m}^{c}={\left(\frac{\sigma_{1}}{F_{m}^{c}}\right)}^{2}={\left(\frac{\sigma_{1}}{F_{1}^{c}}\right)}^{2}\geq 1,\sigma_{1}<0$$

where $d_{f1}^{T}$, $d_{f1}^{C}$, $d_{f2}^{T}$, $d_{f2}^{C}$ represent the damage state variables of fibers under different modes, $F_{1}^{T}$, $F_{2}^{T}$ represent the ultimate tensile stress values in the fiber’s 1-direction and 2-direction, $F_{1}^{C}$, $F_{2}^{C}$ represent the ultimate compressive stress in the fiber’s 1-direction and 2-direction, $S_{12}$, $S_{13}$, $S_{23}$ represent the ultimate shear stress values in the three directions, $d_{m}^{T}$, $d_{m}^{C}$ represent the damage state variables of the matrix under different odes, $F_{m}^{T}$, $F_{m}^{C}$ represent the ultimate tensile stress and ultimate compressive stress values of the matrix material, $\sigma_{ij}$ represents the stress components at the integration points during the calculation process. Table 5 lists the parameters related to finite element analysis, some of which are referenced from the previous literature [22]. Subsequent finite element analysis validation confirms that these parameters fully meet the requirements for the finite element simulations in this study, with the standard deviation of each parameter being below 5%.

## 4. Results and Discussion

### 4.1. Hygrothermal Aging Experiment and Finite Element Analysis

The moisture absorption curve exhibits a trend similar to the results reported in other studies [26,27]. The moisture absorption process can be divided into three stages. In the initial stage, the weight gain rate of the specimen increases linearly with the square root of time, with rapid mass growth, and the effect of temperature on the absorption rate is significant. In the intermediate stage, the moisture absorption process of the specimen tends to stabilize, corresponding to the first change in the slope of the absorption curve. At this point, the diffusion rate of water molecules can be calculated using Equation (24). As time continues to increase, the weight gain rate of the specimen gradually slows down and approaches equilibrium, eventually reaching a state of moisture saturation with minimal mass changes. Experimental results show that mass absorption increases with the square root of time and eventually stabilizes, consistent with Fick’s law [33].

(24) $$D=\pi {\left(\frac{h}{{4M}_{m}}\right)}^{2}{\left(\frac{M_{2}-M_{1}}{\sqrt{t_{2}}-\sqrt{t_{1}}}\right)}^{2}$$

where *h* represents the thickness of the plate; *M_m_* represents the saturated moisture absorption rate; *M*_1_ and *M*_2_ represent the moisture absorption rates at times *t*_1_ and *t*_2_.

**Figure 4:**
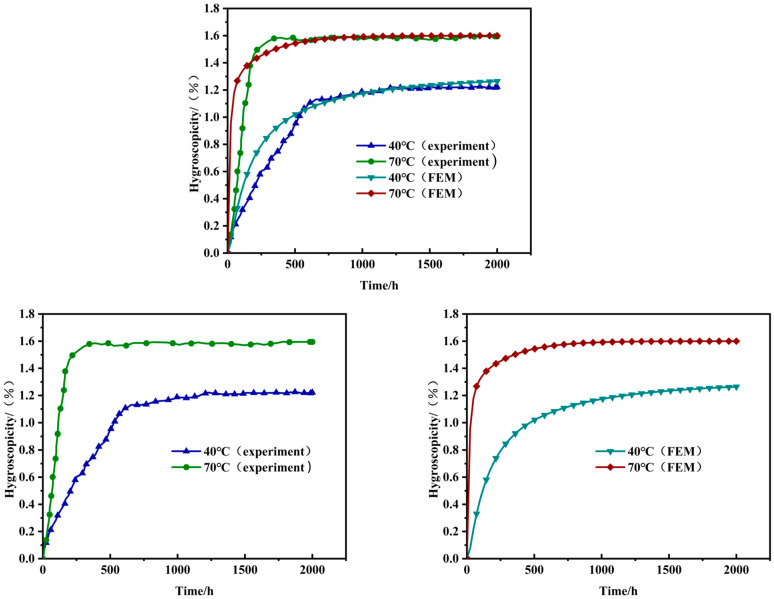
Moisture absorption curve of woven composite panels.

Under a 40 °C water bath, the maximum weight gain rate is approximately 60–70% of that under a 70 °C water bath. It takes about 766 h to reach saturation at 40 °C, while it takes only about 250 h at 70 °C. This is because temperature affects the mobility of water molecules. According to Equation (24), the moisture diffusion rates are 3.46184 × 10^−6^ mm^2^/s (40 °C water bath) and 1.40046 × 10^−5^ mm^2^/s (70 °C water bath), with the diffusion rate at 40 °C being approximately 24% of that at 70 °C. This indicates that temperature variation has a significant effect on the moisture diffusion rate of three-dimensional four-directional composites. This can be seen from the image presented in Figure 4 and the data in Table 6. Comparing the experimental and finite element simulation moisture absorption curves, the results are highly consistent. Under the same water bath conditions, the slope differences between the experimental and simulation curves are minimal, and the saturated moisture absorption rates are nearly identical, especially the maximum weight gain rates under different water bath environments; for the 40 °C and 70 °C water bath environments, the final moisture absorption rates from the finite element simulations differ from the experimental results by 3.477% and 0.317%, respectively. This has important implications for calculating stresses caused by moisture expansion.

**Figure 5:**
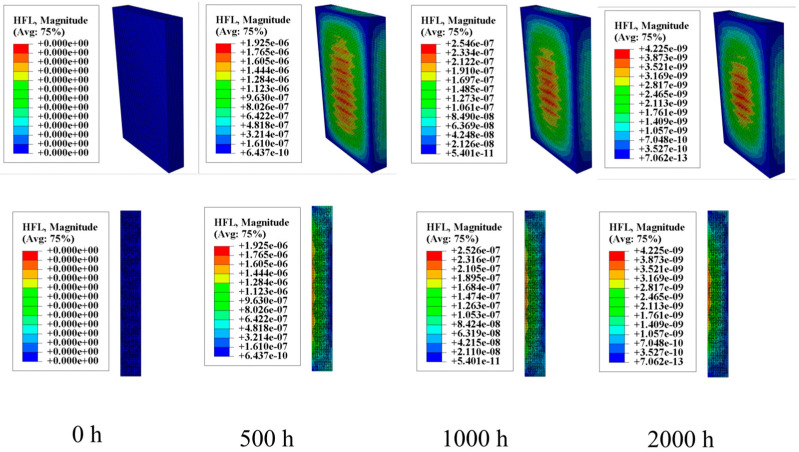
Moisture diffusion process.

To more accurately analyze the internal stress caused by humidity and temperature fields, finite element analysis was conducted using Abaqus software. The equivalence between “mass diffusion” and “heat diffusion” was rigorously validated in the preceding discussion. Therefore, the “heat diffusion” module was employed to simulate the diffusion of water molecules within the specimen. As shown in Figure 5, the humidity field concentration within the specimen gradually increases with extended moisture absorption time. The sectional contour plots illustrate the specific process of water molecules gradually diffusing from the surface into the interior of the specimen.

**Figure 6:**
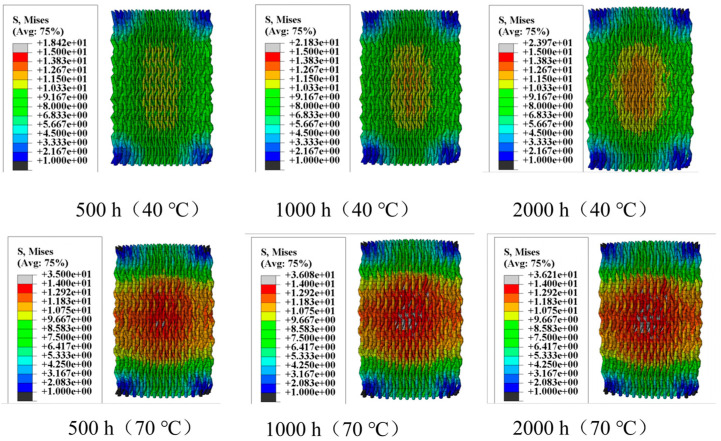
Moisture stress in fiber bundles.

Figure 6 illustrates the moisture stress generated in the fiber bundles within a humidity field. The distribution of moisture stress inside the specimens varies significantly due to differences in hygrothermal aging time and temperature. In the humidity field, carbon fibers absorb almost no moisture, but the epoxy resin matrix is highly sensitive to moisture absorption. Moreover, higher temperatures increase the activity of water molecules, accelerating moisture diffusion. Therefore, when the hygrothermal aging time is constant, the moisture stress in a 70 °C water bath is significantly greater than that in a 40 °C water bath. After 500 h of hygrothermal aging, the maximum moisture stress in a 40 °C water bath is 18.42 MPa, mainly concentrated around 10 MPa. In contrast, in a 70 °C water bath, the maximum moisture stress increases to 35 MPa, with stress near the center of the specimen reaching about 15 MPa. Similar patterns can be observed when comparing the moisture stress distribution at other aging times. When the hygrothermal aging temperature is constant, the moisture stress increases over time, with temperature playing a more significant role in promoting stress.

**Figure 7:**
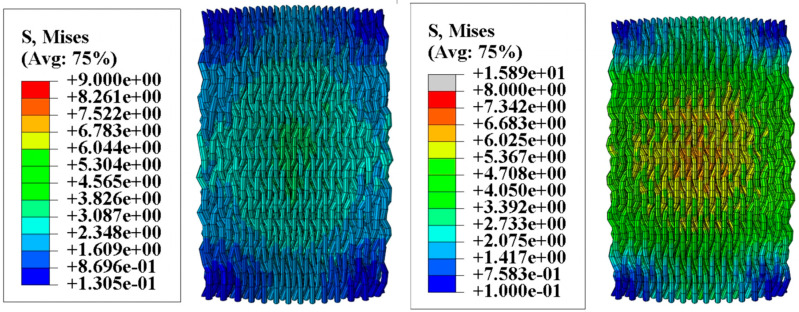
Thermal stress in fiber bundles.

**Figure 8:**
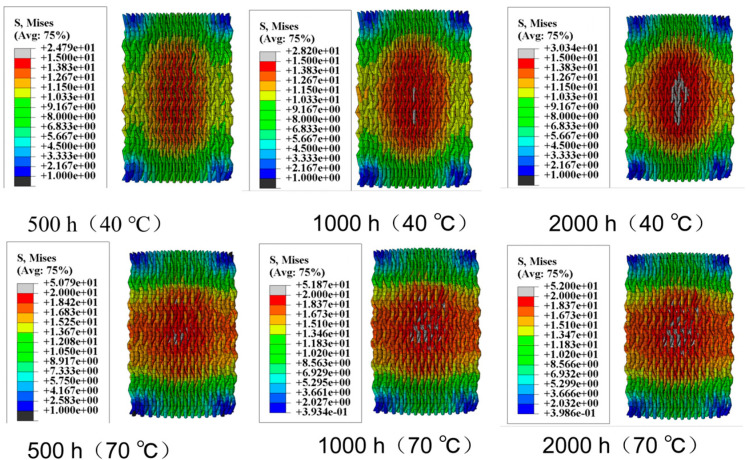
Internal stress in fiber bundles.

Figure 7 shows the thermal stress induced by the thermal field. When the water bath temperature is 40 °C, the maximum thermal stress is 9 MPa; when the temperature increases to 70 °C, the maximum thermal stress rises to 15.89 MPa. By superimposing the moisture stress and thermal stress, the complete internal stress distribution within the specimen can be obtained, as shown in Figure 8. Figure 8 visually demonstrates the residual stress distribution: the higher the hygrothermal aging temperature and the longer the time, the greater the residual stress. The magnitude of the residual stress directly affects the subsequent physical damage strength of the material, a phenomenon that has been confirmed in actual compression experiments. Stress contour maps under hygrothermal environments can accurately predict the stress distribution in different parts of the material, identify stress concentration points, and analyze local stress concentration phenomena. Due to differences in the coefficients of thermal expansion between the matrix and fiber bundles, as well as the characteristics of the three-dimensional four-directional structure, the stress distribution in hygrothermal environments significantly differs from that in laminated materials, exhibiting pronounced non-uniformity.

### 4.2. Compression Experiment and Finite Element Analysis

**Figure 9:**
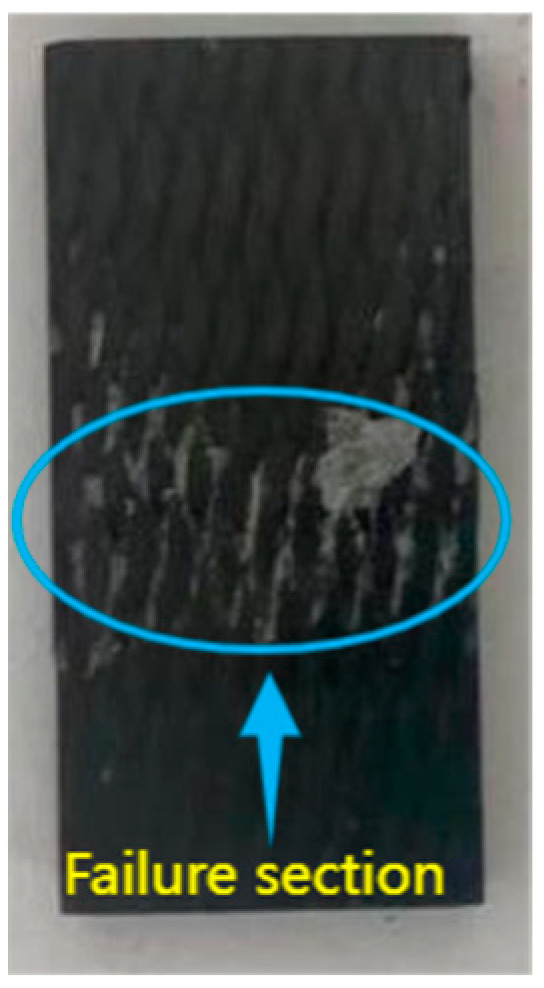
Compressive failure cross-section of three-dimensional four-directional composites.

After the hygrothermal test, the specimens were subjected to compressive loads until failure, and the fracture morphology after compression failure is shown in Figure 9. At the initial stage of compression, no sound was emitted, and no significant deformation was observed. After a short period of compression, the specimen emitted a loud noise and subsequently failed. The fracture after compression was perpendicular to the axial direction, which is consistent with the characteristics of brittle failure observed in the experiment.

At room temperature, the ultimate stress of the three-dimensional four-directional braided material is 357.35 MPa. Table 7 lists the ultimate stresses and strength reduction effects under different hygrothermal treatment conditions. In a hot and humid environment, the reduction in ultimate stress and strength shows a clear temperature dependency. In a 40 °C water bath, as the duration of the moist heat aging increases, the material’s stress gradually decreases, exhibiting a continuous degradation trend. In contrast, in a 70 °C water bath, the rate of decrease in ultimate stress significantly accelerates, indicating that the rise in temperature accelerates the degradation of the material’s performance. Han et al. [34] conducted compression tests on three-dimensional woven composite materials under different temperature conditions and concluded that the compressive performance of the material gradually decreases as the temperature increases; additionally, higher temperatures lead to the softening of the epoxy resin matrix, affecting the bond between the resin and the fibers. This conclusion aligns with the results of Han et al.’s study on the compressive performance of three-dimensional woven composite materials in high-temperature environments. This aligns with the moisture absorption and weight gain phenomenon observed in hygrothermal aging tests: at higher temperatures, water molecules move more vigorously, causing greater damage to the matrix and the matrix fiber interface, while the impact is relatively smaller at lower temperatures. Under the same water bath temperature, the duration of hygrothermal aging also significantly affects the ultimate stress of the specimens. The longer the hygrothermal aging time, the lower the compressive strength of the material and the greater the impact. Observing the compressive strength of the material after 500 h, 1000 h, and 2000 h of hygrothermal aging, it can be seen that shorter aging times result in higher compressive strength. This is because longer hygrothermal aging times cause more severe internal structural damage to the material. After the matrix absorbs water, the compressive strength further decreases, and the connection between the matrix and fibers suffers greater damage with prolonged aging time. Therefore, hygrothermal aging time has a significant impact on the compressive performance of three-dimensional four-directional braided composites, a conclusion consistent with other research findings [35].

**Figure 10:**
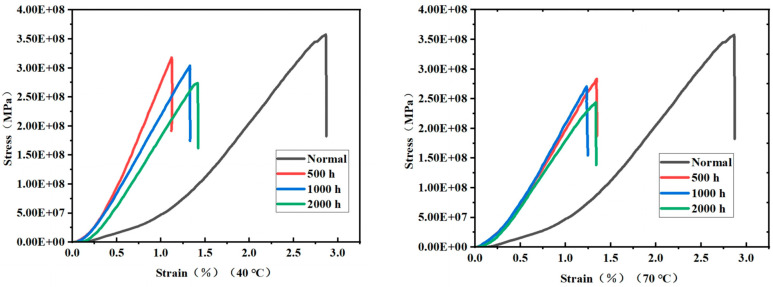
Stress–Strain curve after compression in a 40 °C and 70 °C water bath.

Figure 10 show the stress–strain curves of the material. After hygrothermal treatment, the strain corresponding to the ultimate stress is smaller than under room temperature conditions. After reaching the ultimate stress peak, the curve exhibits a rapid linear drop, which is caused by the fibers fracturing under ultimate conditions, resulting in instantaneous brittle failure.

**Figure 11:**
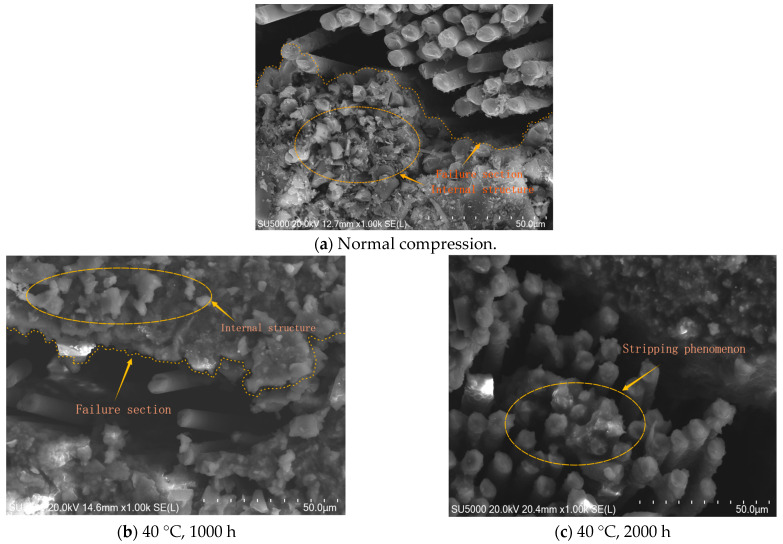

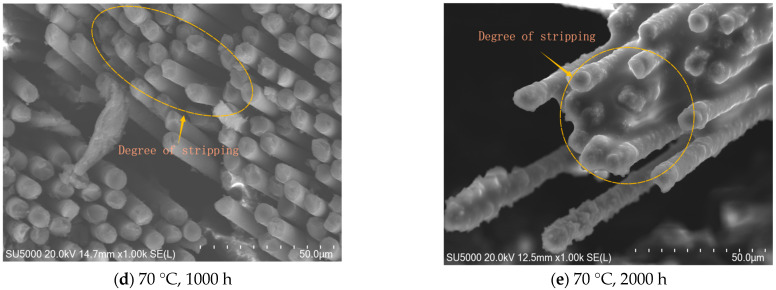
Scanning Electron Microscopy (SEM) image of the internal structure after compression failure. (**a**) Section after compression failure at normal temperature, (**b**) After 40 °C, 1000 h water bath treatment, (**c**) After 40 °C, 2000 h water bath treatment, (**d**) After 70 °C, 1000 h water bath treatment, (**e**) After 70 °C, 2000 h water bath treatment.

As shown in Figure 11, different hygrothermal conditions have significantly distinct impacts on the internal structure. The degree of matrix delamination from the fiber bundles varies, leading to different levels of epoxy resin protection for the fiber bundles. After undergoing hygrothermal aging in a 40 °C water bath for 1000 h, a significant amount of resin remains adhered to the fiber surface after compression failure. However, the resin matrix interface shows signs of chemical degradation caused by the hygrothermal environment. When the aging duration increases to 2000 h, chemical degradation intensifies, and the delamination between the resin and fibers becomes more severe. After treatment in a 70 °C water bath for 1000 h, only a small amount of resin adheres to the fiber bundle surface, with delamination and chemical degradation being the most pronounced. The temperature of the hygrothermal environment and the aging time have a significant impact on the hydrolysis process of epoxy resin. According to studies [35,36,37,38,39], the water absorption of epoxy resin gradually increases with prolonged hygrothermal aging time. The mechanism of this phenomenon can be attributed to the combined effects of high temperature and humidity, which cause water to penetrate into the resin and trigger a series of changes. Specifically, the penetration of water leads to the rearrangement of molecular chains in the epoxy resin, resulting in water absorption and swelling of the resin. After water enters the epoxy resin structure, it disrupts the cross-linked structure of the molecular chains, leading to a significant decline in the mechanical and physical properties of the resin. Water absorption not only causes volumetric expansion of the resin but may also alter its physical properties, such as increasing expansibility and softening. Ultimately, the strength and service life of three-dimensional woven composites are reduced due to the degradation of the epoxy matrix, and this phenomenon has been further validated in this study through scanning electron microscopy. Based on Figure 11, it can be concluded that hygrothermal aging affects the material’s strength, altering its ultimate compressive strength. The hygrothermal environment has a significant impact on the matrix, while the fiber bundles exhibit minimal moisture absorption and demonstrate negative thermal expansion at this temperature. The hygrothermal environment does not alter the material’s three-dimensional four-directional internal structure, thus maintaining the failure mode unchanged.

**Figure 12:**
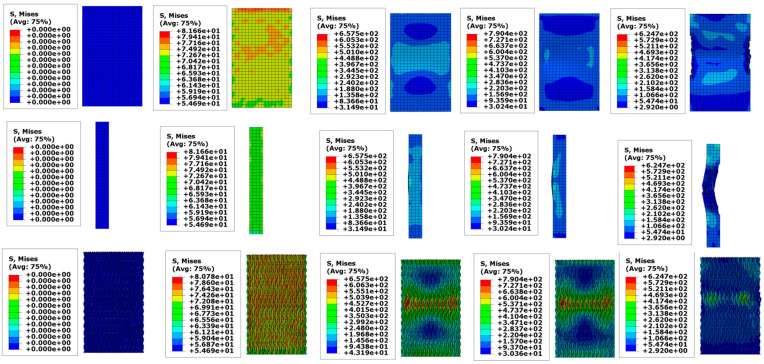
Specimen compression process.

Figure 12 illustrates the compression process of three-dimensional four-directional braided composites. The contour plot indicates that during the axial compression experiment, the specimen gradually yields and ultimately fails, with the fracture plane being perpendicular to the axial direction. This observation aligns with the experimental results.

Under a 40 °C water bath environment, the ultimate stress obtained from finite element simulation decreased to 250.48 MPa, with a 9.2% error compared to the experimental value. At 70 °C, the ultimate stress further decreased to 239.86 MPa, with only a 2.4% error from the experimental data.

**Figure 13:**
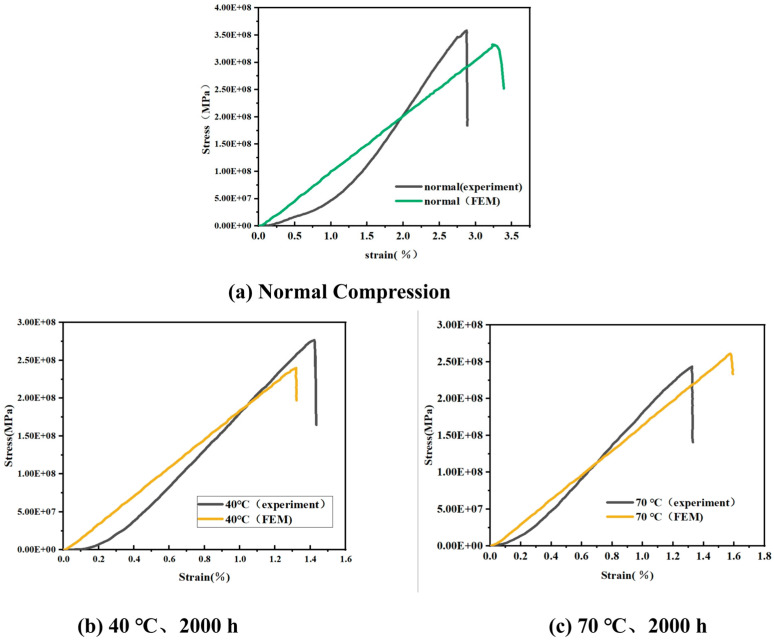
Comparison of simulated stress–strain curves with experimental data by finite element analysis. (**a**) At normal temperature, (**b**) After 40 °C, 2000 h water bath treatment, (**c**) After 70 °C, 2000 h water bath treatment.

As shown in Figure 13b,c, the stress–strain curves obtained from finite element analysis are similar in overall shape to the experimental results, with minimal differences in the slope. This indicates that the model demonstrates high reliability in a hygrothermal environment and meets the accuracy requirements for engineering applications.

Under normal conditions, the ultimate stress from finite element simulation is 330.07 MPa, with a 7.6% error compared to the experimentally measured value. However, in the strain range of 0 to 0.2, discrepancies are observed between the simulated and experimental stress–strain curves. These discrepancies may result from the mismatch between the assumptions of the model and the complex structure of the actual material. In the finite element modeling process, this study primarily considered the reinforcing effect of fibers on the overall structure and the three-dimensional four-directional fabric characteristics while neglecting interfacial effects between the matrix and fiber bundles as well as the development of potential microscopic damage. However, when the strain exceeds 0.2, both the finite element and experimental curves exhibit a consistent linear growth trend, with the error in ultimate stress remaining within 10%. These results indicate that the finite element model adopted in this study can accurately reflect the mechanical behavior of the material under different environmental conditions to a certain extent.

Comprehensive analysis shows that the hygrothermal environment significantly affects the mechanical properties of composite materials, as evidenced by the reduction in ultimate stress and changes in strain response. The finite element simulation results provide a reasonable basis for meeting the accuracy requirements of engineering applications. However, further optimization of the model is required in future research to reduce deviations in the low strain range.

## 5. Conclusions

This study investigates the effects of hygrothermal environments on the compressive performance of three-dimensional four-directional braided composites through hygrothermal aging and compression experiments. Numerical calculations of internal stresses induced by hygrothermal conditions were conducted using the finite element method (FEM). The following key conclusions were drawn:

- Hygrothermal aging duration and environmental temperature jointly influence the compressive performance of the material. As aging time increases and temperature rises, the degree of performance degradation becomes significantly more pronounced, showing a clear pattern of deterioration.
- Temperature accelerates the diffusion of moisture by increasing the activity of water molecules, thereby playing a dominant role in performance degradation caused by hygrothermal conditions. The hygrothermal environment has a significant impact on the epoxy resin matrix but a minimal effect on the fibers. Consequently, the internal structure of the three-dimensional four-directional braided composite remains fundamentally unchanged.
- Finite element simulations reveal that expansion stress caused by the moisture field is greater than thermal expansion stress, leading to localized stress concentrations within the material. These localized stress concentrations significantly affect the mechanical properties of the material and represent a critical mechanism for performance degradation under hygrothermal conditions.

Finally, this study analyzes the aging behavior of three-dimensional four-directional braided composites under hygrothermal conditions, revealing the significant impact on their compressive performance, particularly the stress induced by hygrothermal coupling affecting material strength. The study provides theoretical and data support for composite material design, long-term reliability evaluation, and quality control. Future research could focus on further exploring the long-term effects of various extreme conditions on the performance of three-dimensional four-directional composites, with a potential diversification of failure modes in the specimens.

## Figures and Tables

**Table 1:** Main parameters of experimental materials.

	Properties
Measure	50 mm × 25 mm × 5 mm
Braiding Angles	15°
Fabric structure	three-dimensional four-directional
Yarn fineness	800 tex
Pitch length	41.0 ± 1.0 mm
Pitch width	11.0 ± 0.5 mm
Overall fiber volume content	(60 ± 3)%
Fiber filling factor	75%

**Table 2:** Geometrica parameters of fiber bundles. [28].

*α* (°)	*λ* (g/m)	*ρ* (g/cm^3^)	*W* (mm)	*h* (mm)	*V_f_* (%)
15	0.8	1.8	0.9465	1.7662	60

**Table 3:** Basic Thermal Parameters of Resin Matrix and Carbon Fibers.

	Specific Heat Capacity	Thermal Conductivity	Coefficient of Thermal Expansion
Carbon fiber	710 (J/kg·k)	4.92 (W/m·K)	−4.84343 × 10^−7^/°C
Epoxy resin	1.89 (J/g·°C) (40 °C)	0.18 (W/m·K) (40 °C)	25 × 10^−6^/°C (40 °C)
	1.97 (J/g·°C) (70 °C)	0.27 (W/m·K) (70 °C)	32.5 × 10^−6^/°C (70 °C)

**Table 4:** Comparison of units for heat conduction and humidity diffusion parameters.

Peculiarity	Heat Conduction	Humidity Diffusion
Field variable	Temperature	Relative humidity
density	1 kg/m^2^	1 kg/m^2^
Conductivity	Thermal conductivity (W/m·k)	Wet diffusion coefficient (mm^2^/s)
Dilate	Coefficient of thermal expansion/°C	Humidity expansion coefficient/% H_2_O
Specific heat	1 J/(kg·°C)	1 J/(kg·°C)

**Table 5:** Material parameters related to finite element analysis.

Parameter Name	Carbon Fiber	Epoxy Resin
Young’s modulus, *E*1/GPa	230	2.5
Young’s modulus, *E*2 = *E*3/GPa	15	2.5
Poisson’s ratio, *μ*12 = *μ*13	0.28	0.35
Poisson’s ratio, *μ*23	0.4	0.35
Shear modulus, *G*12 = *G*13/GPa	20	1.28
Shear modulus, *G*23/GPa	5.36	1.28
Longitudinal tensile strength, $F_{1}^{T}$/MPa	4900	57.2
Longitudinal compressive strength, $F_{1}^{C}$/MPa	1470	100
Transverse tensile strength, $F_{2}^{T}$ *=* $F_{3}^{T}$/MPa	69	57.2
Transverse compressive strength, $F_{2}^{C}$ *=* $F_{3}^{C}$/MPa	100	100
Shear strength, *S*_12_ = *S*_13_/MPa	90	90
Shear strength, *S*_23_/MPa	90	90
Density, *ρ* (cm^2^)	1.79	1.19

**Table 6:** Saturation time and maximum weight gain.

	Moisture Saturation Time at 40 °C Water Bath	Moisture Saturation Time at 70 °C Water Bath	Maximum Weight Gain Rate at 40 °C Water Bath	Maximum Weight Gain Rate at 70 °C Water Bath
Experimental Results	766 h	250 h	1.22166%	1.59482%
Simulation Results	792 h	288 h	1.26414%	1.59987%

**Table 7:** Ultimate stress after compression in different hygrothermal environments.

	Ultimate Stress (40 °C)	Cut Down	Ultimate Stress (70 °C)	Cut Down
500 h	319.433 Mpa	10.611%	284.452 Mpa	20.400%
1000 h	304.431 Mpa	14.809%	272.035 Mpa	23.874%
2000 h	275.760 Mpa	22.832%	245.848 Mpa	31.202%

## Data Availability

The data presented in this study are available on request from the corresponding author due to privacy.

## References

[1] Ansar M., Xinwei W., Chouwei Z. (2011). Modeling strategies of 3D woven composites: A review. Compos. Struct..

[2] Friedrich K., Almajid A.A. (2012). Manufacturing Aspects of Advanced Polymer Composites for Automotive Applications. Appl. Compos. Mater..

[3] Wang Z., Xie H., Luo Q., Li Q., Sun G. (2021). Optimizaition for formability of plain woven carbon fiber fabrics. Int. J. Mech. Sci..

[4] Wang Y., Liu Z.-G., Wei Y.-C., Li Z.-j., Yi Y.-P., Wang Y.-B. (2020). Novel processing technology and mesoscopic geometric modeling of a new three-dimensional (3D) braided composite and the study on its longitudinal mechanical properties. Compos. Struct..

[5] Jing X., Cheng Z., Teng X., Yang X., Shi D. (2020). Reconstruction of meso-structure and numerical simulations of the mechanical behavior of three-dimensional four-directional braided ceramic matrix composites. Ceram. Int..

[6] Zhang D., Zheng X., Wang Z., Wu T., Sohail A. (2020). Effects of braiding architectures on damage resistance and damage tolerance behaviors of 3D braided composites. Compos. Struct..

[7] Carey J., Fahim A., Munro M. (2004). Design of braided composite cardiovascular catheters based on required axial, flexural, and torsional Rigidities. J. Biomed. Mater. Res. Part B-Appl. Biomater..

[8] Gong L., Gao X., Yang H., Liu Y., Yao X. (2018). Design on the driveshaft of 3D 4-Directional carbon fiber braided composites. Compos. Struct..

[9] Liu J., Ge H., Chen J., Wang D., Liu H. (2012). The preparation of emulsion type sizing agent for carbon fiber and the properties of carbon fiber/vinyl ester resin composites. J. Appl. Polym. Sci..

[10] Simar A., Gigliotti M., Grandidier J.-C., Ammar-Khodja I. (2018). Decoupling of water and oxygen diffusion phenomena in order to prove the occurrence of thermo-oxidation during hygrothermal aging of thermosetting resins for RTM composite applications. J. Mater. Sci..

[11] Bouazza M., Zenkour A.M. (2018). Free vibration characteristics of multilayered composite plates in a hygrothermal environment via the refined hyperbolic theory. Eur. Phys. J. Plus.

[12] Tsenoglou C.J., Pavlidou S., Papaspyrides C.D. (2006). Evaluation of interfacial relaxation due to water absorption in fiber-polymer composites. Compos. Sci. Technol..

[13] Abdel-Magid B., Ziaee S., Gass K., Schneider M. (2005). The combined effects of load, moisture and temperature on the properties of E-glass/epoxy composites. Compos. Struct..

[14] Johar M., Chong W.W.F., Kang H.S., Wong K.J. (2019). Effects of moisture absorption on the different modes of carbon/epoxy composites delamination. Polym. Degrad. Stab..

[15] Yu K., She Y., Chen J., Cai X., Wu Y. (2024). Pressureless Immersion of Epoxy Resin-Filled Cracks in Faulted Rock Materials. Materials.

[16] Amoushahi H., Goodarzian F. (2018). Dynamic and buckling analysis of composite laminated plates with and without strip delamination under hygrothermal effects using finite strip method. Thin-Walled Struct..

[17] Kumar S.B., Sridhar I., Sivashanker S. (2008). Influence of humid environment on the performance of high strength structural carbon fiber composites. Mater. Sci. Eng. A-Struct. Mater. Prop. Microstruct. Process..

[18] Kawai M., Yagihashi Y., Hoshi H., Iwahori Y. (2013). Anisomorphic constant fatigue life diagrams for quasi-isotropic woven fabric carbon/epoxy laminates under different hygro-thermal environments. Adv. Compos. Mater..

[19] Chakraverty A.P., Mohanty U.K., Mishra S.C., Biswal B.B. (2017). Effect of Hydrothermal immersion and Hygrothermal Conditioning on Mechanical Properties of GRE Composite. IOP Conf. Ser. Mater. Sci. Eng..

[20] Benkhedda A., Tounsi A., Bedia E.A.A. (2008). Effect of temperature and humidity on transient hygrothermal stresses during moisture desorption in laminated composite plates. Compos. Struct..

[21] Magazzu A., Marcuello C. (2023). Investigation of Soft Matter Nanomechanics by Atomic Force Microscopy and Optical Tweezers: A Comprehensive Review. Nanomaterials.

[22] Fang G., Liang J., Lu Q., Wang B., Wang Y. (2011). Investigation on the compressive properties of the three dimensional four-directional braided composites. Compos. Struct..

[23] Zhu H., Du X.-b., Li D.-s., Jiang L. (2022). Investigation of parameterized braiding parameters and loading directions on compressive behavior and failure mechanism of 3D four-directional braided composites. Compos. Struct..

[24] Liao D., Gu T., Liu J., Chen S., Zhao F., Len S., Dou J., Qian X., Wang J. (2024). Degradation behavior and ageing mechanism of E-glass fiber reinforced epoxy resin composite pipes under accelerated thermal ageing conditions. Compos. Part B Eng..

[25] Chen D., Meng M., Sun X., Guan M., Yang B., Xiao S. (2024). Effects of Hygrothermal Aging on the Flexural Properties of Cross-Ply and Angle-Ply CFRP Composite Laminates. Fibers Polym..

[26] Zhang Y., Li H., Yan S., Wang X., Guan Y., Du C., Jiang L., Zhai J. (2024). Experimental Analysis of the Low-Velocity Impact and CAI Properties of 3D Four-Directional Braided Composites after Hygrothermal Aging. Materials.

[27] Guan Y., Yan S., Chen X., Zhang Y., Wang X., Li H., Zhao Y., Zhai J. (2024). Analysis of Residual Post-Impact Compressive Strength of Composite Laminates Under Hygrothermal Conditions. Appl. Compos. Mater..

[28] Wang X., Li H., Zhang Y., Guan Y., Yan S., Zhai J. (2024). Compressive Failure Characteristics of 3D Four-Directional Braided Composites with Prefabricated Holes. Materials.

[29] Sun X., Sun C.J. (2004). Mechanical properties of three-dimensional braided composites. Compos. Struct..

[30] Korta J., Mlyniec A., Uhl T. (2015). Experimental and numerical study on the effect of humidity-temperature cycling on structural multi-material adhesive joints. Compos. Part B-Eng..

[31] Geng G., Ma X., Geng H., Wu Y. (2018). Effect of Thermal Cycles on the Thermal Expansion Behavior of T700 Carbon Fiber Bundles. Chem. Res. Chin. Univ..

[32] Hashin Z.F. (1981). Fatigue failure criteria for unidirectional fiber composites. ASME J. Appl. Mech..

[33] Ray B.C. (2006). Temperature effect during humid ageing on interfaces of glass and carbon fibers reinforced epoxy composites. J. Colloid Interface Sci..

[34] Han W.F., Li D.S., Jiang L. (2020). High-temperature properties and failure mechanism of 3D six-directional braided composites under out-of-plane compression. Polym. Compos..

[35] Wang Y., Meng Z., Zhu W., Wan B., Han B., Cai G., Yin X., Bai Y. (2021). Hygrothermal aging behavior and aging mechanism of carbon nanofibers/epoxy composites. Constr. Build. Mater..

[36] Boubakri A., Elleuch K., Guermazi N., Ayedi H.F. (2009). Investigations on hygrothermal aging of thermoplastic polyurethane material. Mater. Des..

[37] Dong Z., Wu G., Zhu H., Zhao X.-L., Wei Y., Qian H. (2020). Flexural behavior of seawater sea-sand coral concrete–UHPC composite beams reinforced with BFRP bars. Constr. Build. Mater..

[38] Prolongo S.G., Gude M.R., Ureña A. (2012). Water uptake of epoxy composites reinforced with carbon nanofillers. Compos. Part A Appl. Sci. Manuf..

[39] Wang M., Xu X., Ji J., Yang Y., Shen J., Ye M. (2016). The hygrothermal aging process and mechanism of the novolac epoxy resin. Compos. Part B Eng..

